# Cascade genetic counseling and testing in hereditary syndromes: inherited cardiovascular disease as a model: a narrative review

**DOI:** 10.1007/s10689-023-00356-x

**Published:** 2024-01-06

**Authors:** Laura A. Grutters, Imke Christiaans

**Affiliations:** Department of Genetics, University of Groningen, University Medical Center Groningen, Hanzeplein 1, P.O. Box 30.001, Groningen, 9700 RB The Netherlands

**Keywords:** Cascade genetic testing, Genetic counseling, Inherited cardiovascular diseases, Familial hypercholesterolemia, Hereditary cancer syndromes, Family communication

## Abstract

Inherited cardiovascular diseases cover the inherited cardiovascular disease familial hypercholesterolemia and inherited cardiac diseases, like inherited cardiomyopathies and inherited arrhythmia syndromes. Cascade genetic counseling and testing in inherited cardiovascular diseases have had three decades of academic attention. Inherited cardiovascular diseases affect around 1–2% of the population worldwide and cascade genetic counseling and testing are considered valuable since preventive measures and/or treatments are available. Cascade genetic counseling via a family-mediated approach leads to an uptake of genetic counseling and testing among at-risk relatives of around 40% one year after identification of the causal variant in the proband, with uptake remaining far from complete on the long-term. These findings align with uptake rates among relatives at-risk for other late onset medically actionable hereditary diseases, like hereditary cancer syndromes. Previous interventions to increase uptake have focused on optimizing the process of informing relatives through the proband and on contacting relatives directly. However, despite successful information dissemination to at-risk relatives, these approaches had little or no effect on uptake. The limited research into the barriers that impede at-risk relatives from seeking counseling has revealed knowledge, attitudinal, social and practical barriers but it remains unknown how these factors contribute to the decision-making process for seeking counseling in at-risk relatives. A significant effect on uptake of genetic testing has only been reached in the setting of familial hypercholesterolemia, where active information provision was accompanied by a reduction of health-system-related barriers. We propose that more research is needed on barriers -including health-system-related barriers- and how they hinder counseling and testing in at-risk relatives, so that uptake can be optimized by (adjusted) interventions.

## Introduction

Cascade genetic testing is a systematic process designed to identify at-risk relatives within families with a known disease-causing genetic variant. This methodology is particularly pertinent for inherited diseases where preventive measures and/or treatments are available, such as inherited cardiovascular diseases and hereditary cancer syndromes. We provide a narrative review focusing on the former: cascade genetic counseling and testing within inherited cardiovascular diseases and we will discuss what is known about its uptake in different types of inherited cardiovascular diseases. Inherited cardiovascular diseases can be split into:

- Inherited cardiovascular diseases, mainly being familial hypercholesterolemia (FH), in which the high cholesterol gives rise to premature atherosclerosis which can cause myocardial and cerebral infarctions and sudden death at young age.
- Inherited cardiac diseases (ICDs) that have a disease mechanism within the heart. Within this group we can make a distinction between the inherited cardiomyopathies, which present with structural abnormalities of the myocardium and lead to heart failure and potentially fatal ventricular arrhythmias, and the inherited arrhythmia syndromes that mainly affect the functioning of the electrical circuit of the heart and present with ventricular arrhythmias leading to syncope, cardiac arrest or sudden death.

Inherited cardiovascular diseases have many common denominators: they can all give rise to sudden death, symptoms can present at young age mostly in adulthood, but sometimes in childhood, they mostly have autosomal dominant inheritance with variable disease expression and incomplete age-dependent penetrance and they are medically actionable. In many ways these inherited cardiovascular diseases are comparable with inherited cancer syndromes. For all these late-onset medically actionable diseases guidelines have recommended predictive genetic testing for relatives due to available preventive or risk mitigating actions and they can be seen as Tier 1 genetic conditions in the CDC classification system from the United States.

Within the Netherlands we have almost three decades of experience with cascade genetic testing for ICDs within families. This review therefore presents an in-depth exploration of cascade genetic counseling and testing and its uptake among at-risk relatives within the cardio(vascular)genetics framework, which can serve as an illustrative model for inherited cancer syndromes. Our focus is primarily on cascade genetic counseling and, when appropriate, genetic testing. We do not delve into clinical cardiac evaluations in at-risk relatives in the absence of genetic testing. The overarching aims of this narrative review are to provide insights into the successes and constraints of cascade genetic testing in at-risk relatives, the barriers identified in information provision to at-risk relatives by probands, the barriers that prevent at-risk relatives from seeking genetic counseling and testing and to extrapolate our findings from inherited cardiovascular disorders to other late-onset medically actionable hereditary diseases, like hereditary cancer syndromes. To lay the groundwork for this narrative review, we first provide background information for the different types of ICDs, discuss their prevalence to illustrate the magnitude of the issue and describe current cardiogenetic care. Since familial hypercholesterolemia has had different predictive testing settings in many countries, we provide more information on these settings and findings on uptake in FH in a separate paragraph.

## Current cardiogenetic care in inherited cardiac diseases (inherited cardiomyopathies and inherited arrhythmia syndromes)

Cascade genetic testing within families with ICDs commenced in the Netherlands in 1996, coinciding with the advent of diagnostic DNA-testing for conditions such as long QT syndrome (LQTS) and hypertrophic cardiomyopathy (HCM) [1]. Since then, cascade genetic testing has emerged as a vital and cost-effective tool for identifying at-risk relatives in cardiogenetics [2–7]. It enables relatives with the disease-causing variant to undergo early treatment and prevention efforts, resulting in health benefits and ultimately reducing the cardiovascular burden in the population. Relatives who test negative for the disease-causing variant do not need, or can be released from, ongoing clinical surveillance, as can their offspring.

A recent review by Hayesmoore et al. (2023) reported the indications for genetic testing, the core genes involved for different ICDs and disease prevalence estimates [8]. The prevalence of HCM and dilated cardiomyopathy (DCM) in the general population is estimated to range from 1 in 500 to 1 in 200–250 individuals. Arrhythmogenic right ventricular cardiomyopathy is estimated to have a disease prevalence ranging from 1 in 5,000 to 1 in 1,000 individuals, with certain populations, such as in Greece and the Netherlands, exhibiting higher prevalence rates due to the presence of founder variants. LQTS is estimated to affect 1 in 3,000 to 1 in 2,000 individuals, while the prevalence of Brugada syndrome is estimated to be 1 in 5,000 to 1 in 2,000 individuals [8]. Catecholaminergic polymorphic ventricular tachycardia (CPVT) has a disease prevalence of 1 in 10,000 [9]. Combining these prevalence estimates of ICDs -without taking into account higher prevalence of phenotype negative genotype positive individuals- results in an overall prevalence ranging between 1 in 211 and 1 in 90 individuals in the general population. When extrapolating these numbers to the Dutch population of 17.5 million people, there are an estimated 82,833 to 194,250 people affected by ICDs in the Netherlands. Within the Dutch population, approximately 760 new families with a genetic variant predisposing to ICDs are identified each year [local numbers extrapolated to national numbers 2022]. These numbers clearly illustrate the magnitude of the issue.

As most ICDs have an autosomal dominant inheritance pattern and are rarely present *de novo*, all first-degree relatives have a 50% risk of carrying the causal variant, and cascade genetic counseling and testing is provided to at-risk relatives. In the Netherlands, genetic counseling is offered to these relatives through a family letter written by the genetics department and distributed by the proband (the first affected person in a family to have a DNA-test) [10]. This family-mediated approach is followed in many Western countries, but in some (Western) countries DNA-diagnostics is offered to both probands and at-risk relatives within the field of cardiology, with or without involvement of a genetics department or genetic counselor. Although cardiac guidelines on ICDs recommend a multidisciplinary team for proper genetic counseling and diagnostics, it is currently still unclear how and if relatives are informed about an ICD in different parts of the world [11].

Nevertheless, current clinical genetic practice in the Netherlands relies on the proband to inform at-risk relatives when a (likely) pathogenic genetic variant is identified. To establish a standardized approach to family communication, in 2019 the Dutch Clinical Genetics Society (VKGN) released a guideline for informing relatives about inherited diseases that is especially relevant for ICDs and hereditary cancer syndromes [10]. This guideline recommends discussing with patients about the way of informing relatives, allowing options for direct contact of relatives by the genetics department. However, in clinical practice in most families relatives are informed by the patient. As a result, approximately 3800 at-risk relatives undergo genetic counseling for ICDs in the Netherlands each year [local numbers extrapolated to national numbers 2022]. This counseling of at-risk relatives supports them in making informed decisions about cascade genetic testing, preventive measures, timely treatment and reproductive options. It also provides carriers of the causal variant with regular monitoring by a cardiologist and treatment or preventive options if needed. Examples of interventions include early detection of cardiomyopathy through echocardiography, pharmacological treatment to reduce heart failure symptoms and implantation of an implantable cardioverter-defibrillator for the prevention of sudden death. In LQTS and CPVT, a beta-blocker is prescribed as a preventive measure against ventricular arrhythmias and sudden cardiac death. In addition, non-carriers can be reassured and do not need cardiac evaluation and genetic testing of offspring. Second- and further-degree relatives can subsequently be counseled and, if desired, tested [12]. Cascade genetic testing in ICDs has been shown not to negatively affect quality of life and to increase perceived personal control even in asymptomatic relatives who are identified as carriers of the causal variant [13].

In summary, ICDs affect around 1–2% of the population worldwide, with current cascade genetic testing mostly offered via a family-mediated approach and early identification via genetic testing shown to positively impact patients’ health and wellbeing.

## Uptake of cascade genetic counseling and testing in ICDs

From a genetic counseling point of view, it is important that all relatives be informed about an inherited disease in their family with the aim that they can make an informed decision. From a public health point of view, for inherited diseases like ICDs for which there are treatment and preventive options, it is important to identify as many relatives with the causal variant as possible. However, both aims -informed decision-making and prevention- can only be achieved with optimal uptake of genetic counseling and genetic testing [14]. The uptake of genetic counseling is the percentage of at-risk relatives attending genetic counseling. The uptake of genetic testing is the percentage of at-risk relatives having a genetic test for the familial causal variant. Here, we focus on the uptake of cascade genetic counseling when employing the family-mediated approach, where relatives are informed by the proband. Thereafter, we describe the barriers to probands supplying at-risk relatives with information and the barriers to at-risk relatives seeking genetic counseling.

Worldwide, the first studies on the uptake of counseling in ICDs came from the Netherlands. Christiaans et al. (2008) informed at-risk relatives, by means of a family letter disseminated through the proband, about the aims, opportunities and possible drawbacks of cascade genetic testing. They showed that uptake of genetic counseling was 39% one year after the detection of the causal variant in 97 families with HCM [14]. Uptake of genetic counseling did not differ significantly with the probands’ or relatives’ gender, nor with the young age (< 18 years) of the relative or with a family history positive for sudden cardiac death. Furthermore, uptake in first-degree relatives was 40.4%. In second-degree relatives, who were directly eligible for cascade genetic testing when the connecting first-degree relative had died, uptake was 27.5% [14]. In addition, the study of Van der Roest et al. (2009) included 8 probands with LQTS with a causal variant and 7 with HCM with a causal variant to inform 12 and 42 relatives, respectively, using a family letter distributed by the proband [15]. They highlighted a notable difference in response between these two conditions: 83% of relatives from LQTS families sought cardiologic and/or genetic consultation within a mean follow-up period of two years compared to 40% in the HCM families. Miller et al. (2013) evaluated the uptake of cascade genetic testing by relatives in a cohort of 40 probands with cardiomyopathy and a causal variant within a four year window in the United States [12]. Probands were asked to share a family letter with their relatives. Genetic testing was indicated for 213 (140 first and 73 s-degree relatives) relatives. In the four year period, 72 (51%) first-degree and 12 (16%) second-degree relatives underwent genetic testing. First-degree relatives were more likely to undergo genetic testing, but the number of living affected relatives did not affect uptake of genetic testing [12]. Burns et al. (2016) reported an overall uptake of cascade genetic testing within LQTS families of 60% in four years using a family-mediated approach, although some relatives received cardiac follow-up because of LQTS in their family and were (also) informed by their cardiologist [16]. A multicenter study reporting on uptake of cardiac/genetic testing in relatives of children diagnosed with HCM or LQTS mostly reported uptake rates per family instead of per relative, showing an uptake of any form of testing in 90% of families of genetically confirmed index cases (a family was counted as having uptake if at least one relative had testing), but 26% of relatives was mentioned not to have had any testing, being similar for HCM and LQTS families [17]. Unfortunately, this study did not clearly report on uptake per relative nor did it specify the time period of testing in relatives. To have any comparison the study by Christiaans et al. (2008) mentioned a similar uptake (87%) per family of adult HCM patients within a multiyear follow-up, but a one year uptake in relatives of 40%, meaning that a high follow-up per family does not have to mean a high uptake among all relatives [14]. A recent trial on uptake in different types of ICDs by Van den Heuvel (2022) showed a one-year uptake of 38% in the cohort using the family mediated approach for informing relatives and a similar uptake in the previous year in a non-study cohort, showing that uptake rates in ICDs have not improved since the first reports from 2008 and not since the Dutch guideline on informing relatives [18].

In 2020 Van den Heuvel et al. reported the long-term uptake in the families with a causal variant previously reported by Christiaans and Van der Roest. In these 115 families uptake by 717 eligible relatives (598 first- and 119 s-degree relatives) was evaluated over a median period of 16 years [19]. Overall, 41% and 46.3% of eligible relatives attended genetic counseling after the first year and the second year, respectively. After a median follow-up period of 16 years, the uptake of cascade genetic counseling was 60%: 64% among first- and 41% among second-degree relatives. Uptake of genetic counseling was observed more frequently in first-degree relatives, female relatives, primary arrhythmia syndromes, relatives with manifest disease, relatives without children and families with sudden cardiac death in first-degree relatives < 40 years [19]. Van den Heuvel et al. (2020) and Christiaans et al. (2008) have documented that 98.4% and 99% of *counseled* relatives, respectively, opted to pursue genetic testing, possibly indicating that individuals typically seek genetic counseling only after they have already decided to undergo genetic testing and this observation aligns with our experience from counseling sessions in clinical practice [14, 19].

In sum, uptake of cascade genetic counseling and testing in cardiomyopathies is around 40% one year after identification of the causal variant in the proband, remains far from complete on the long-term with one study reporting an uptake of 60% after 15 years among eligible at-risk relatives when using the family-mediated approach. For arrhythmia syndromes uptake can be slightly higher in the first years.

## Barriers in information provision to at-risk relatives by probands

Below we will delineate potential threats to information provision to at-risk relatives due to barriers perceived by probands and challenges associated with the family-mediated approach. Research on the barriers perceived by probands with inherited diseases is mostly performed in the field of oncogenetics and barriers can be divided into four categories (Table 1). *Knowledge barriers* such as not knowing which relatives are at risk, *attitudinal barriers* such as thinking that relatives prefer not to know and guilt, *social reasons* such as the social and emotional consequences in family context and, lastly, *practical barriers* such as having no contact details from relatives [20–22]. Although probands generally understand the importance of sharing information with relatives, these barriers demonstrate that informing relatives through the proband, as is done in the family-mediated approach, poses several threats [23]. The most important is that this approach may result in information not being shared, being incompletely shared or not being shared in a timely way, thereby contributing to limited uptake. At-risk relatives will only be able to decide on genetic counseling and testing if they are made aware of the possibility. For example, in hereditary cancer, up to 30% of relatives are not aware of their familial cancer risk [24]. Certain interventions, for example enhanced telephone support for probands in informing at-risk relatives, have been effective in increasing uptake of genetic counseling (26% versus 21% after 18 months), although the uptake reported in this randomized controlled trial, even with the intervention of telephone support, was still below average [25]. It also seems difficult to effectively influence the sharing of genetic test results with relatives. A recent review by Ballard et al. (2023) summarizes interventions designed to increase the likelihood that probands shared relevant genetic health information with their appropriate relatives [26]. The authors conclude that knowledge, motivation and self efficacy were not increased in any intervention although two studies reported that the intervention was well received by patients and health professionals. In oncogenetics, a review by Young et al. (2022) gathered interventions that provided tailored extra appointments with genetic counselors using specific communication techniques including motivational interviewing [27]. These interventions showed a wide range in the percentage of informed relatives ranging from 54 to 95.5%. Only one out of six included studies in the review by Young et al. (2022) documented a significant effect on the percentage of informed relatives by an intervention using follow-up telephone contacts with the proband dedicated to the subject of informing relatives versus no intervention in a retrospective cohort (75% versus 34%). To note, within this study distribution of a family letter directly to relatives via the medical team was also offered to probands. This study documented an increased proportion of relatives seeking contact with clinical genetic department in the intervention group (60% versus 30% within two years of follow-up) [28]. The other five studies did not show a significant effect of the intervention on uptake. These studies suggest, but do not conclusively show, that tailored genetic counseling with additional follow-up can sometimes increase the proportion of informed relatives and uptake of genetic counseling, but the effect is often minimal or non significant or can be related to the effect of time instead of intervention [27]. Therefore, when using the family-mediated approach, considering family dynamics hindering information dissemination and the characteristics of the target population is essential.

**Table 1:** Barriers in information provision to at-risk relatives by probands [21, 22]

Barriers* in probands	Examples
Knowledge	Not knowing which relatives are at risk, poor recall of genetic information
Attitudinal	Thinking that relatives prefer not to know, guilt
Social	The social and emotional consequences in family context such as bad relationships and family conflicts
Practical	Having no contact details from relatives, relatives living abroad

*Several barriers intertwine

## Directly contacting at-risk relatives

One possible solution for the pitfalls we have described would be an active approach to inform relatives. Direct contact with relatives by health care professionals has been suggested as a way to increase uptake of genetic counseling and testing by increasing the number of informed relatives. However, privacy laws have complicated this process because directly contacting at-risk relatives without probands consent is prohibited in most Western countries. Consequently, there is only limited literature on interventions that enable more direct contact with relatives in the field of ICDs and other hereditary conditions. Interventions include sending family letters directly to the relatives or initiating telephone conversations with relatives by health care professionals. Direct contact with at-risk relatives has an inconclusive effect on uptake on genetic counseling and testing in oncogenetics as was reported by a review study of Frey et al. (2022) [29]. Dissemination of information to relatives directly by the medical team through telephone calls, letters or emails was done in 16 studies and reported to increase cascade genetic counseling uptake to 63% and testing to 53%. Duration of time since probands’ genetic testing was not documented. Direct contact with relatives via a telephone call by a health care professional was demonstrated most effective with genetic counseling uptake of 84% and testing of 61% [29]. There were several limitations to the study designs, which were carried out in rather small cohorts using diverse methodologies and without control groups, making it difficult to interpret findings. For example, in hereditary colorectal cancer, distribution of a family letter directly to at-risk relatives yielded high uptake rates, but the study participants including the relatives were identified from a national cancer registry, which indicates that they may already have been aware of their increased cancer risk [30]. Another example can be found in the small study by Sermijn et al. (2016) which showed that sending a family letter directly to at-risk relatives 6 to 12 months after they were informed by the proband led to an increase in uptake, but no control group was assessed, so the increased uptake rates could also be attributed to the passage of time [31]. In the field of ICDs, a recent randomized controlled trial by Van den Heuvel et al. in 2022 demonstrated that informing relatives through a more active and tailored approach, including sending a family letter directly to at-risk relatives, did not have a significant effect on the uptake of genetic counseling (37%) and testing within one year compared to the family-mediated approach (38%) [18]. The direct contact approach was well appreciated by probands and relatives. However, the fact that all relatives in the intervention cohort were informed did not have an influence on uptake of genetic counseling.

To conclude, while previous interventions focusing on optimizing the informing of relatives through the proband and on contacting relatives directly have been proven to be well appreciated by the proband, the effect on uptake of genetic counseling and testing was only minimally. Focus should probably be on barriers that hinder informed at-risk relatives to seek genetic counseling and testing.

## Barriers hindering informed at-risk relatives from seeking genetic counseling and testing

So even though barriers in information provision to at-risk relatives by probands could be overcome by directly contacting relatives, uptake of genetic counseling remains incomplete. A study by Bednar et al. (2020) reported that probands with hereditary cancer mentioned that 80% of their untested first-degree relatives were aware of the variant in the family, but only 11% intended to have genetic testing in the next one to six months [32]. So far, additional investigation into the barriers that hinder at-risk relatives from actively seeking counseling services have identified several factors which can be divided into four categories (Table 2). This research has primarily been carried out within families with hereditary cancer, and information has often been identified indirectly through probands or other relatives attending genetic counseling and not by questioning non-attending at-risk relatives [20–22, 33]. Research on these barriers specifically in ICDs is limited, and the data on their effect on uptake is clouded by intertwining barriers among probands and at-risk relatives. In LQTS, socioeconomic status, anxiety, and depression have been suggested to negatively influence family communication and the uptake of cascade genetic testing [16].

**Table 2:** Barriers hindering at-risk relatives from seeking genetic counseling and testing [21, 22]

Barriers* in at-risk relatives	Examples
Knowledge	Misconceptions about the disease and/or the risk, misunderstanding of how to obtain a referral
Attitudinal	Preferring not to know, rejection of responsibilities, fear of discrimination in the context of marriage or employment
Social	Anxiety, stage of life
Practical	High costs of predictive DNA-testing, fear of consequences for obtaining disability or life insurance, language barrier

*Several barriers intertwine

Among HCM families (probands and at-risk relatives), Khouzam et al. (2015) identified health-care provider recommendations, specific familial factors (higher uptake when first-degree relatives were diagnosed with HCM and having a genetic mutation identified in the family) and a more favorable view of testing as the strongest indicators of uptake of genetic testing in an analysis combining both probands and relatives [34]. As knowledge is the main factor hindering an increase in uptake, the ability of relatives to understand the complex information provided by the proband or health care professional is a point for investigation and consideration. Only limited literature is available on relatives’ perspectives on the family letter. Although uptake of genetic counseling is recognized to be increased after implementation of a family letter, a study by Dheensa et al. suggested that the letters are perceived as lengthy and complicated and cause anxiety among relatives [35]. These findings were, however, not confirmed by Zordan et al., who found that the length and content of the family letters did not significantly affect patients’ understanding, feelings and intention to contact a health care professional [36]. When looking at the target population of at-risk relatives, uptake of genetic counseling and testing differs among ICDs. In inherited arrhythmias like LQTS the uptake of counseling seems to be higher than in HCM, and it has been suggested that this is related to the severity of health complaints in the proband, which are more often directly life threatening, and at younger age in LQTS and thus the perceived risk of sudden death in those eligible for testing, may be higher in inherited arrhythmia families than in most cardiomyopathy families [1, 15]. In HCM, Khouzam et al. (2015) and Christiaans et al. (2008) did not find a significant association between family history of sudden cardiac arrest/death and uptake of genetic testing, regardless of the relationship with the proband, but the larger uptake study of Van den Heuvel et al. did show that sudden death in a first degree relative had an effect on uptake but only when this occurred before the age of 40 years [14, 19, 34].

To conclude, several barriers that hinder cascade genetic counseling and testing in at-risk relatives have been identified, mainly in inherited forms of cancer. However, the precise extent to which these factors play a role i the decision-making process about seeking counseling is currently unknown.

## Uptake in familial hypercholesterolemia

FH is an autosomal dominantly inherited cardiovascular disorder with an estimated prevalence of 1 in 250 [37]. Carriers of a causal variant have a high cholesterol and develop premature atherosclerosis and atherosclerosis-related events like myocardial infarctions and cerebral infarctions. Atherosclerosis and related events can be prevented by lifestyle modifications (e.g., diet and excersice) and medication (statins). Because of the preventive potential in FH, a unique government-subsidized, cost-free cascade genetic testing program for FH was introduced in the Netherlands in 1994. Within this program, at-risk relatives were actively approached after written consent from the proband. A specialized nurse carried out home visits for written consent, blood sampling for genetic testing and collection of personal and family data. This strategy yielded a participation rate of 90% within the first 5 years, leading to the identification of an estimated 3.22% of the total FH population in the Netherlands in 1999 [6, 38]. Active cascade genetic testing for FH seemed highly acceptable among relatives. Van Maarle et al. showed that < 5% of relatives were troubled by being actively approached [39]. Moreover, participation in the genetic testing program did not negatively impact the quality of life of relatives who participated [23, 40]. Up to 2014, > 28,000 patients (5,000 probands and 23,000 relatives), an estimated 41.49% of the total FH population in the Netherlands, were identified [41]. However, since 2014, the active approach is prohibited due to new regulations within the healthcare system, and the family-mediated approach as for ICDs has now become the standard. Since then, participation rates have decreased from an average of 8 relatives per proband to 2–3 per proband [37, 41].

The advantage of the previous active approach was that it removed several barriers for relatives. First of all, they were actively informed about the genetic condition in the family directly by the health care professional. Subsequently, counseling and testing was performed during a home visit and was free of any costs. An additional notable exception for FH, instigated by government policy, was the legal requirement that individuals with FH applying for disability or life insurance had to be accepted at standard rates if their LDL-c level was below 4.0 mmol/l and they had no additional cardiovascular risk factors [42]. This circumvented the perceived practical barrier of fear of consequences in obtaining disability or life insurance by at-risk relatives that seems to hinder uptake of cascade genetic counseling and testing in ICDs. The beliefs that it is impossible to obtain an insurance as a variant carrier is a widespread misconception. In daily practice it has been shown that fewer than 5% of variant carriers tested in a predictive setting experienced difficulties obtaining disability or life insurance in the Netherlands [43].

A well-established, free, genetic testing program for FH has also been available in Norway since 1998 via a family-mediated approach and a national programme has started in Northern Ireland in 2000. These programs led to the identification of an estimated 51% of the Norwegian FH population by 2020 and 17% of the Northern Ireland FH population by 2018 [6, 44]. In contrast, identification of the FH population in other parts of the UK without well-established programs ranged from 4 to 9% in 2018 [6]. Uptake rates for cascade genetic testing in the US, also without a well-established program, have been reported to be 4–12% [45]. The uptake of cascade genetic testing for FH in the United States is low due to a number of barriers such as the lack of a centralized and coordinated cascade testing program, the inability of health care providers to directly contact relatives due to the Health Insurance Portability and Accountability Act Privacy Rule and complex family dynamics [46].

Compared to the cascade genetic testing approach for ICDs, cascade genetic testing for FH in the Netherlands was initially initiated and sponsored by the government, provided at no cost and implemented with a proactive strategy, including home visits by a nurse. This groundbreaking method resulted in remarkable participation rates and overall satisfaction. Furthermore, this approach effectively addressed various barriers arising from the Dutch healthcare systems’ structure, such as offering genetic testing without requiring deductible payments, ensuring no impact on disability or life insurance and eliminating the need to consult a general practitioner for a referral.

## A comparsison with hereditary cancer syndromes

The combined prevalence of hereditary cancer syndromes is probably higher than that of ICDs (without FH), which is also reflected in the number of counseled probands and relatives. In the Netherlands, a variant predisposing to hereditary cancer syndromes is identified in approximately 1100 families and approximately 5900 at-risk relatives undergo genetic counseling for hereditary cancer syndromes each year (in comparison to 760 families and 3800 at-risk relatives in ICDs, respectively) [local numbers extrapolated to national numbers 2022]. As in ICDs and FH, most first-degree relatives in hereditary cancer syndromes face a 50% risk of inheriting the familial disease-causing variant, and a family-mediated approach is the standard of care in cascade genetic testing. Cascade genetic testing empowers relatives to access proactive measures like organ-specific screening, risk-reduction surgeries and prompt therapeutic interventions to minimize the chances of cancer development and improve overall quality of life. Similar to ICDs, the identification of causal genes, exemplified by breast cancer genes *BRCA1* and *BRCA2*, commenced in the 1990s, enabling the practice of family cascade genetic testing in oncogenetics. A review by Frey et al. (2022) included 38 studies of hereditary breast and ovarian cancer and hereditary colorectal cancer in which the family-mediated approach was used. The average uptake of cascade genetic counseling was 35% and cascade genetic testing was 36%, with uptake highest in first-degree relatives and females. Direct dissemination of information to relatives by the medical team through telephone calls, letters or emails was done in 16 studies and has been reported to increase cascade genetic counseling uptake to 63% and testing to 53%. Duration of time since probands’ genetic testing was not documented [29]. Many, other interventions have been examined which mostly did not have an effect on uptake (see paragraph on barriers). However, small cohorts, diversity in methodology and the absence of a direct comparison of the family-mediated and direct approach make it difficult to interpret these findings. Uptake rates in the field of oncogenetics align with those observed in the context of ICDs. An active approach is suggested as an opportunity to increase uptake, however the studies conducted showed no or limited effect on uptake and have notable limitations, mainly the absence of a control group. In conclusion, similar uptake rates and barriers seem to play a roll in the fields of oncogenetics and cardiogenetics.

## Discussion

This narrative review provides an overview of cascade genetic counseling and testing and its uptake and barriers among at-risk relatives in the context of inherited cardiovascular diseases (inherited cardiac diseases (ICDs) and familial hypercholesterolemia). The uptake among at-risk relatives attending cascade genetic counseling and testing in ICDs is below desired: one study reporting 40% one year after identification of a disease-causing variant in the proband, increasing to 60% after 15 years. Uptake is highest in first-degree relatives and can be slightly higher in arrhythmia syndromes. Almost all counseled relatives opt to pursue genetic testing, supporting that individuals typically seek genetic counseling only after they have decided to undergo genetic testing.

These findings align with uptake rates among relatives at-risk for hereditary cancer syndromes. To improve uptake rates, many interventions have been introduced, mainly in supporting probands in informing their relatives. However, this seems to have no or little effect on uptake. Direct contact with relatives has also been studied. Only in the setting of FH, in which active information provision was accompanied by a reduction of health-system-related barriers, was a significant effect on uptake reached. Therefore, only providing information to at-risk relatives has minimal impact on increasing the uptake of genetic counseling. Barriers relating to ‘informed’ relatives making the step to counseling play an important role. First of all, it is questionable how well these relatives are informed. The ability of relatives to understand the complex information provided by the genetics department via the proband must be considered. Little is known about preferences with respect to the optimal amount and type of information and about the potential impact of different digital communication methods on at-risk relatives’ engagement in counseling. No studies have investigated the impact of repeated distribution of the family letter on uptake in the long-term. Nor is it known how the information provided (e.g., family letter) influences the intention to seek genetic counseling and how it is best tailored to the needs of at-risk relatives. In addition, the content of information and the use of incorporating techniques such as message framing to study the effect on intention to seek genetic counseling has not been studied. Most at-risk relatives who do not want genetic testing make this choice without consulting a genetic counselor. At present, many at-risk relatives are not tested, for many reasons, including faulty assumptions and misconceptions. Research on the barriers that impede at-risk relatives from seeking genetic counseling has revealed knowledge, attitudinal, social and practical barriers, but the precise extent to which these factors play a role in the decision-making process about seeking counseling is currently unknown. Therefore, it is essential to further identify and elucidate the barriers that hinder counseling and testing in at-risk relatives that might be influenced by interventions. For instance, the influence of barriers arising from the structure of the healthcare system, such as the need to personally pay excess insurance deductible costs or the need to consult the general practitioner to obtain a referral, on uptake of cascade genetic counseling and testing are not well studied. In this light, the illustrative model of FH has shown that tackling health-care-system-related barriers might be a promising way of increasing uptake.

## Conclusion

In summary, the family-mediated approach of informing relatives of ICD patients leads to a relatively low percentage of uptake of cascade genetic counseling and testing. This uptake is in need of improvement, both from a genetic counseling and a public health point of view. The experience with FH has shown that a direct-contact approach to informing relatives can have a positive effect on uptake, and is feasible and well tolerated, but only when it is incorporated within a broader approach that also addresses other health-care-system-related barriers. It seems worthwhile to put more effort into unravelling the barriers hindering relatives’ participation in cascade genetic counseling and testing, working to identify areas where novel interventions can be implemented to enhance uptake. Optimal uptake is essential for both informed decision-making and prevention in the hereditary diseases where prevention and/or treatment are available like ICDs and hereditary cancer syndromes.
